# Mouse aortic biomechanics are affected by short-term defective autophagy in vascular smooth muscle cells

**DOI:** 10.1186/s12576-022-00829-1

**Published:** 2022-03-11

**Authors:** Dorien G. De Munck, Arthur J. A. Leloup, Sofie De Moudt, Guido R. Y. De Meyer, Wim Martinet, Paul Fransen

**Affiliations:** Laboratory of Physiopharmacology, University of Antwerp, Universiteitsplein 1, 2610 Antwerp, Belgium

**Keywords:** Autophagy, Vascular smooth muscle cells, Mouse aorta, Arterial stiffness

## Abstract

The physiology of vascular smooth muscle (VSMC) cells is affected by autophagy, a catabolic cellular mechanism responsible for nutrient recycling. Autophagy-inducing compounds may reverse arterial stiffening, whereas congenital VSMC-specific autophagy deficiency promotes arterial stiffening. The elevated aortic stiffness in 3.5-month-old C57Bl/6 mice, in which the essential autophagy-related gene Atg7 was specifically deleted in the VSMCs (Atg7^F/F^ SM22α-Cre^+^ mice) was mainly due to passive aortic wall remodeling. The present study investigated whether aortic stiffness was also modulated by a shorter duration of autophagy deficiency. Therefore, aortic segments of 2-month-old Atg7^F/F^ SM22α-Cre^+^ mice were studied. Similarly to the older mice, autophagy deficiency in VSMCs promoted aortic stiffening by elastin degradation and elastin breaks, and increased the expression of the calcium binding protein S100A4 (+ 157%), the aortic wall thickness (+ 27%), the sensitivity of the VSMCs to depolarization and the contribution of VGCC mediated Ca^2+^ influx to *α*_1_ adrenergic contractions. Hence, all these phenomena occurred before the age of 2 months. When compared to autophagy deficiency in VSMCs at 3.5 months, shorter term autophagy deficiency led to higher segment diameter at 80 mmHg (+ 7% versus − 2%), normal baseline tonus (versus increased), unchanged IP_3_-mediated phasic contractions (versus enhanced), and enhanced endothelial cell function (versus normal). Overall, and because in vivo cardiac parameters or aortic pulse wave velocity were not affected, these observations indicate that congenital autophagy deficiency in VSMCs of Atg7^F/F^ SM22α-Cre^+^ mice initiates compensatory mechanisms to maintain circulatory homeostasis.

## Background

In physiological conditions, autophagy is a homeostatic process that is found at basal levels in almost all cell types. In stress conditions, however, autophagy can be upregulated to recycle nutrients and to generate energy for cell survival [1]. Due to aging or genetic defects, vascular autophagy may decline, which is linked to many age-associated diseases such as arterial stiffening, atherosclerosis and hypertension [2]. On the other hand, pharmacological induction of autophagy with, for example, spermidine or rapamycin may extend lifespan and delay cardiovascular aging [3, 4]. Natural aging is associated with structural and mechanical changes in the vessel wall such as increased intima–media thickness, vascular remodeling, increased arterial stiffness and inflammation. A phenotype switch from a contractile to a proliferative/synthetic phenotype, altered growth of vascular smooth muscle cells (VSMCs), their migration in the media, as well as changes in the ratio of collagen and elastin promote these age-associated effects. Moreover, with aging, changes in VSMC contractile signaling and cytoskeletal organization lead to altered vascular contractility [5], but the vascular contractile state and phenotype are also affected in pathological conditions such as hypertension [6]. Many of these responses that are characteristic for natural aging also occur with experimentally induced vascular autophagy reduction or stimulation. Activation of autophagy with platelet-derived growth factor can induce a phenotypic switch from a contractile to a synthetic, proliferative VSMC phenotype [7]. Conversely, blocking autophagy via deletion of the essential autophagy gene Atg7 in VSMCs leads to premature stress-induced senescence, increased collagen secretion and enhanced migration [8]. In 3.5-month-old C57BL/6J mice, we demonstrated that autophagy deficiency in the smooth muscle cells affected VSMC contraction and cellular homeostasis with significant effects on vascular reactivity and calcium homeostasis [9]. Moreover, passive aortic stiffness (i.e., the stiffness of the aorta without active VSMC tone) in these mice was significantly increased at higher transmural pressures, which could be attributed to extracellular matrix remodeling [10]. Since in most vascular disorders passive vessel changes occur as a result of previous alterations in endothelial cell (EC) or VSMC physiology, we hypothesize that autophagy disruption in the VSMC of younger mice (2 months old) induces acute alterations in the vasculature. Therefore, in the present study we evaluate the aortic biomechanical parameters and structure in these very young C57BL/6J mice with or without a VSMC-specific deletion of Atg7 in order to test for active and passive modulation of aortic function by short-term autophagy deficiency.

## Methods

### Mice

Mice on a C57BL/6J background expressing Cre recombinase under control of the actin-binding transgelin (SM22α) promoter and homozygous for the Atg7flox (Atg7^F/F^) allele (14 Atg7^F/F^ SM22α-Cre^+^ mice) [9, 10] as well as their wild-type littermates lacking the Atg7 floxed allele (Atg7^+/+^) but expressing SM22α-Cre (14 Atg7^+/+^ SM22α-Cre^+^ mice) were housed in a temperature-controlled room with a 12-h light/dark cycle and had free access to water and normal chow. VSMCs from Atg7^F/F^ SM22α-Cre^+^ mice show typical features of impaired autophagy such defective autophagosome formation under autophagy-stimulating conditions (starvation), lack of LC3 processing, strong accumulation of p62 and decreased levels of ATG12-ATG5, as previously described [8, 9]. For both mouse strains equal amounts of male (*n* = 9) and female (*n* = 5) mice were examined. Because western blot analyses of Atg7^F/F^ SM22α-Cre^+^ aortic tissue did not reveal clear differences in the efficiency of Atg7 gene deletion in male versus female mice, and the arterial stiffness parameter *E*_*p*_ was not different between the two sexes in both Atg7^+/+^ SM22α-Cre^+^ and Atg7^F/F^ SM22α-Cre^+^ animals, we did not further consider mouse gender in the present study.

Experiments were performed when mice reached the age of 2 months. Animals were euthanized with sodium pentobarbital (250 mg kg^−1^, i.p.), followed by perforation of the diaphragm. The thoracic aorta was carefully excised and stripped from adherent tissue. Starting from the diaphragm, the aorta was cut in six 2 mm long segments (TA1–TA5). TA1 and TA2 were mounted in traditional organ baths and TA3 and TA4 were mounted in The Rodent Oscillatory Tension Set-up to study Arterial Compliance (ROTSAC). TA5 was used for western blotting or histochemistry. Atg7^F/F^ SM22α-Cre^+^ and Atg7^+/+^ SM22α-Cre^+^ mice were always dissected in parallel. All experiments were approved by the Ethical Committee of the University of Antwerp.

### Isometric contraction experiments

Vessel segments (2 mm long) were mounted in organ chambers (10 ml), immersed in Krebs–Ringer (KR) solution [containing (in mM): 118 NaCl, 4.7 KCl, 2.5 CaCl_2_, 1.2 KH_2_PO_4_, 1.2 MgSO_4_, 25 NaHCO_3_, 0.025 CaEDTA and 11.1 glucose] at 37 °C and continuously aerated with 95% O_2_–5% CO_2_ as described previously [10, 11]. Segments were gradually stretched and set at a preload of 16 mN for optimal force development [12]. Force development was measured isometrically with a Statham UC2 force transducer (Gould, Cleveland, OH) connected to a Powerlab 8/30 data-acquisition system (AD Instruments, Spechbach, Germany). When necessary, endothelium-derived relaxation by nitric oxide (NO) was prevented by inhibiting NO formation with 300 µM Nω-nitro-L-arginine methyl ester (L-NAME; Sigma-Aldrich) [12]. Depolarization-dependent contractions of vessel segments were elicited by increasing concentrations of KCl (K^+^: 5.9, 10, 15, 20, 25, 30, 40, and 50 mM). The different K^+^ solutions were prepared by replacing NaCl in the KR solution with equimolar amounts of KCl. A Ca^2+^-free environment (0Ca^2+^) was prepared by omitting Ca^2+^ from the KR solution and adding 1 mM EGTA (Sigma-Aldrich) to chelate remaining Ca^2+^ residues. Phasic contractions by the *α*_1_-adrenergic receptor agonist phenylephrine (PE, Sigma-Aldrich) were determined by adding 2 µM PE to the 0Ca^2+^ solution after 3 min [13]. To restore normal conditions hereafter, 3.5 mM Ca^2+^ was added to the 0Ca^2+^ solution, leading to a tonic contraction by PE. The Ca^2+^ channel blocker diltiazem was used at the maximal concentration of 35 µM to determine the contribution of voltage-gated Ca^2+^ channels (VGCCs) to the tonic PE-induced contractions [14]. Relaxation was induced by increasing concentrations of acetylcholine (3 × 10^–9^ − 10^−5^ M) or, when basal NO was inhibited with L-NAME, by the NO-donor diethylamine NONOate (DEANO) (3 × 10^–10^ − 10^−5^ M). Prior to relaxation, pre-contractions were induced by 2 µM PE.

### Rodent oscillatory tension set-up to study arterial compliance (ROTSAC)

Ex vivo vascular stiffness was determined via ROTSAC measurements as previously described [10, 15]. In brief, aortic vessel segments (2 mm long) were mounted in organ chambers (8 ml) between two wire hooks and segments were continuously stretched between alternating preloads to mimic different diastolic and systolic transmural pressures. Transmural pressure was calculated by using the dimensions of the vessel wall and the applied force (Laplace’s law), as described [15]. Vessel segments were continuously stretched between the different calculated diastolic and systolic pressures at a physiological frequency of 10 Hz to mimic the physiological heart rate in mice (600 beats/minute). At any given pressure, calibration of the upper hook allowed calculation of the diastolic and systolic vessel diameter (mm), the compliance (µm/mmHg) and the Peterson modulus (*E*_*p*_). *E*_*p*_ was defined as the pulse pressure [difference between diastolic and systolic pressure (*∆P*), which was 40 mmHg in the present study], multiplied by the diastolic diameter (*D*_0_), and divided by the diameter change (*∆D*) between diastolic and systolic pressure, or *E*_*p*_ = *D*_0_ × *∆P*/*∆D*. For brevity, we will refer to the vessel *E*_*p*_ as stiffness. Contraction and relaxation of vessel segments were elicited as described above.

### Blood pressure, echocardio-parameters and pulse wave velocity (PWV)

One week before killing, peripheral blood pressure was measured with the CODA tail-cuff method as previously described [16]. In brief, a pressure–volume sensor was attached distally to an occluding cuff to the tail of conscious restrained mice for blood pressure recording. Systolic and diastolic blood pressure were measured on three consecutive days, of which the final measurement was used. Next, transthoracic echocardiograms were acquired in anesthetized mice [1.5–2.5% isoflurane v/v (Forene, Abbvie)] using high-frequency ultrasound (Vevo2100, Visualsonics). Heart rate was maintained at 500 ± 50 beats/min and body temperature between 36 and 38 °C. M-mode images were obtained for left ventricular (LV) function evaluation on short axis view, including measurement of interventricular septum (IVS) thickness, LV posterior wall (LVPW) thickness, and LV lumen diameter of three consecutive respiratory cycles. Fractional shortening (FS), ejection fraction (EF), LV mass and stroke volume (SV) were calculated. In vivo arterial stiffness was determined by measuring PWV according to a method developed by Di Lascio et al. [17] in the abdominal aorta of anesthetized mice [1.5–2% isoflurane (Forene Abbvie)] using a high-frequency ultrasound system (Vevo2100, Visualsonics) and a 24-MHz transducer. Briefly, heart rate was maintained at 500 ± 50 beats/min and body temperature between 36 and 38 °C. The aortic diameter (*D*) was measured on 700 frames-per-second B-mode images of the abdominal aorta in EKV imaging mode. Hereafter, pulse wave Doppler tracing was used to measure aortic flow velocity (*V*). The ln(*D*)-*V* loop method was then applied to calculate PWV using Matlab v2014 software (Mathworks).

### Western blot analyses

Vessel segments were lysed in Laemmli sample buffer (Bio-Rad) containing 5% β-mercaptoethanol. Samples were heat-denatured for 5 min and loaded on Bolt 4–12% or 12% Bis–Tris Gels (Life Technologies). After gel electrophoresis, proteins were transferred to Immobilon-FL PVDF membranes (Merck Millipore) according to standard procedures. Membranes were blocked for 1 h in Odyssey Blocking Buffer (LI-COR Biosciences) diluted 1:5 with PBS. After blocking, membranes were probed overnight at 4 °C with primary antibodies (diluted in Odyssey Blocking Buffer), followed by a 1 h incubation with IRDye-labeled secondary antibodies at room temperature. Antibody detection was achieved using an Odyssey SA infrared imaging system (LI-COR Biosciences). The intensity of the protein bands was quantified using Image Studio software. The following primary antibodies were used: mouse anti-LC3B (Nanotools, clone 5F10, 0231-100), rabbit anti-SQSTM1/p62 (Sigma-Aldrich, P0067), rabbit anti-eNOS (sc-654, Santa Cruz), mouse anti P-eNOS (S1177) (612392, BD biosciences) and mouse anti-β-actin (Sigma-Aldrich, A5441). IRDye-labeled secondary antibodies (goat anti-mouse IgG, 926-68070, and goat anti-rabbit IgG, 926-32211) were purchased from LI-COR Biosciences.

### Histology

Vessel segments were fixed in 4% formalin for 24 h and paraffin embedded. Transversal sections were stained with hematoxylin–eosin, orcein or Sirius red to determine wall thickness, elastin and collagen content, respectively. The composition of the aorta was also assessed by immunohistochemistry using the following primary antibodies: anti-collagen type I (Abcam, ab21286), anti-collagen type III (Chemicon, HAB1343), anti-collagen type IV (DAKO, M0785), anti-fibronectin (Abcam, ab2413), anti-vinculin (Invitrogen, 53-9777-80), anti-paxillin (ABCAM, Ab32084) and anti-S100A4 (DACO, A5114). Hereafter, tissue sections were incubated with species appropriate horseradish peroxidase-conjugated secondary antibody (Vector Laboratories), followed by 60 min of reactive ABC (Vector Laboratories). Immunocomplexes were detected with 3,3′-diaminobenzidine or 3-amino-9-ethyl-carbazole (Vector Laboratories). Images were acquired with Universal Grap 6.1 software using an Olympus BX40 microscope and quantified with Image J software.

### Statistics

All data are expressed as mean ± SEM with n representing the number of mice. Statistical analyses were performed using Graphpad Prism software (version 8.3.0). Statistical tests are mentioned in the figure legends. *p* < 0.05 was considered as statistically significant.

## Results

### Defective VSMC autophagy changes aortic biomechanics in a pressure-dependent way

**Fig. 1:**
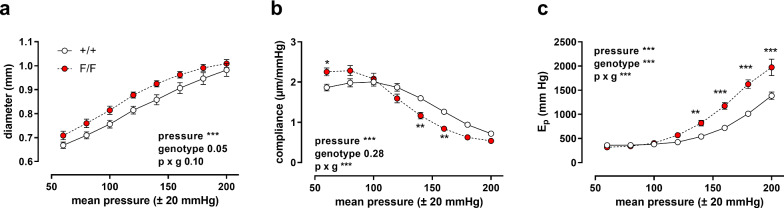
Defective autophagy in VSMCs increases arterial stiffness at high pressures. Pressure-dependency of diastolic diameter (**a**), compliance (**b**) and *E*_*p*_ (**c**) of Atg7^+/+^ SM22α-Cre^+^ (+/+) and Atg7^F/F^ SM22α-Cre^+^ (F/F) aortic segments (*n* = 7). Pulse pressure was always 40 mmHg, hence each mean pressure point is ± 20 mmHg (stretch frequency 10 Hz). Two-way ANOVA with pressure and genotype effect indicated and with Sidak’s multiple comparisons post-test. *p < 0.05 **p < 0.01 ***p < 0.001

**Fig. 2:**
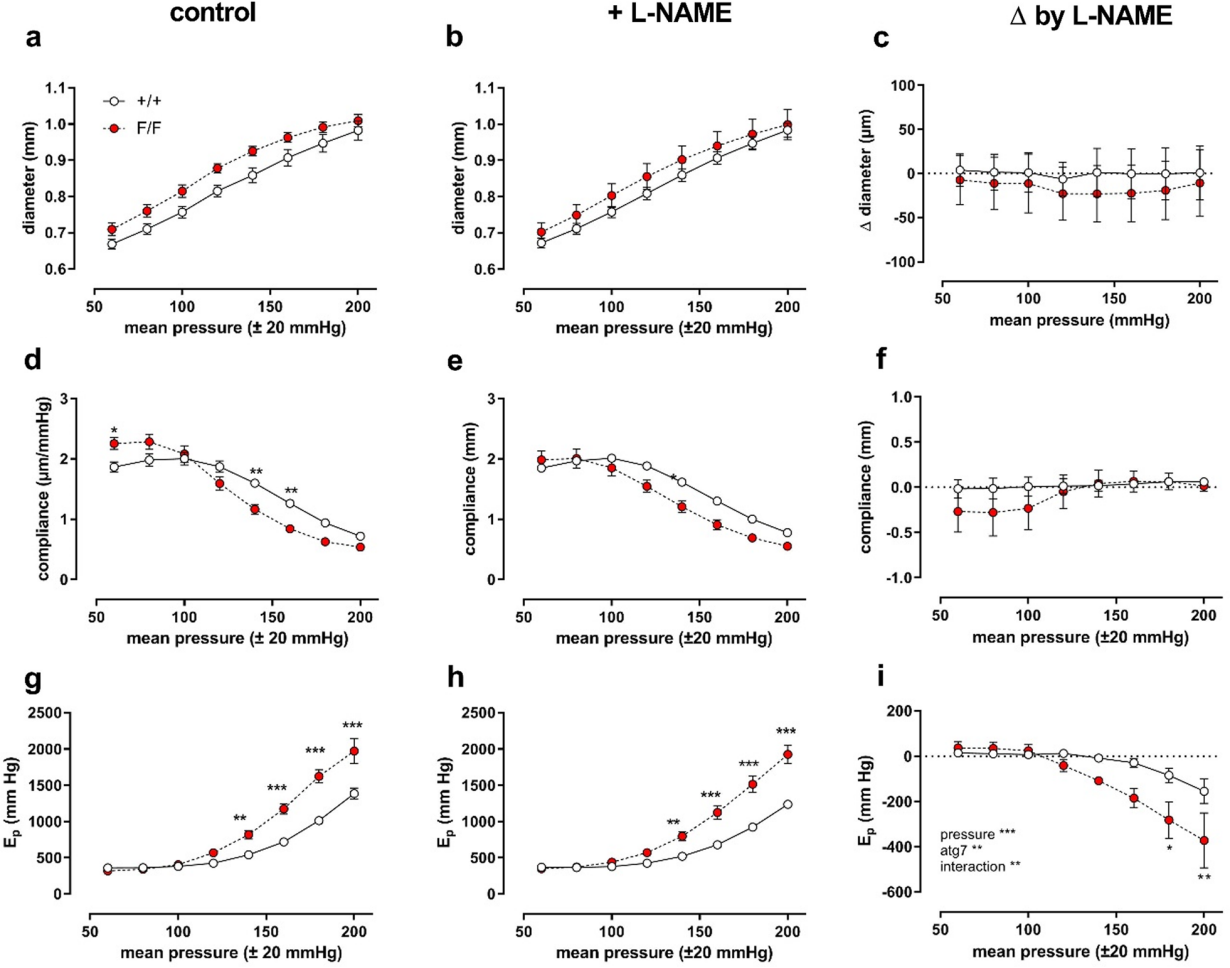
General pressure-dependency of aortic diameter, compliance and stiffness is not dependent on inhibition of eNOS by L-NAME. Defective autophagy in VSMCs increases arterial stiffness at high pressures. Pressure-dependency of diastolic diameter (**a**, **b**, **c**), compliance (**d**, **e**, **f**) and *E*_*p*_ (**g**, **h**, **i**) of Atg7^+/+^ SM22α-Cre^+^ (+/+) and Atg7^F/F^ SM22α-Cre^+^ (F/F) aortic segments (*n* = 7) in the absence (**a**, **d**, **g**) and presence of 300 µM L-NAME (**b**, **e**, **h**). In **c**, **f** and **i** the absolute change of diameter, compliance and *E*_*p*_ induced by L-NAME addition and blocking eNOS is shown as a function of distension pressure. Pulse pressure was always 40 mmHg, hence each mean pressure point is ± 20 mmHg (stretch frequency 10 Hz). Two-way ANOVA with pressure, genotype (atg7) and the interaction effect between both is indicated. Statistical comparison for the different pressure points is calculated with Sidak’s multiple comparisons post-test. *p < 0.05 **p < 0.01 ***p < 0.001

The pressure dependency of the diastolic diameter (*D*_0_), compliance and *E*_*p*_, a vessel diameter-independent measure for arterial stiffness, of aortic segments of Atg7^+/+^ SM22α-Cre^+^ and Atg7^F/F^ SM22α-Cre^+^ mice was determined in baseline (KR) conditions from a mean pressure of 60 mmHg (40–80 mmHg) to a mean pressure of 200 mmHg (180–220 mmHg) in steps of 20 mmHg. With higher distension pressures, aortic diameters increased, and although there were no significant differences between the mouse strains, the diastolic diameter was larger in aortic segments of Atg7^F/F^ SM22α-Cre^+^ mice as compared to control mice (*p* = 0.05) (Fig. 1a). In control mice, compliance was highest at a physiological pressure of 80–120 mmHg, while in aortic segments of Atg7^F/F^ SM22α-Cre^+^ mice compliance was already decreasing at this pressure (Fig. 1b). In addition, at high pressures *E*_*p*_ values of Atg7^F/F^ SM22α-Cre^+^ mouse vessel segments were significantly elevated, indicating higher stiffness (Fig. 1c). Overall, the shape of the pressure curves was not significantly affected by the addition of L-NAME (Fig. 2), except for aortic stiffness at high pressures. This might suggest higher basal NO release in baseline conditions in Atg7^F/F^ SM22α-Cre^+^ mouse aortic segments.

### Defective VSMC autophagy increases aortic stiffness passively

**Fig. 3:**
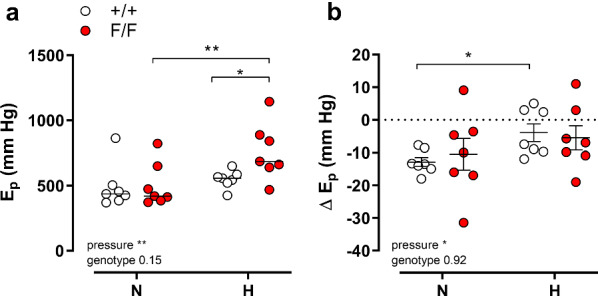
Defective autophagy in VSMCs increases passive arterial stiffness at high pressures. Aortic stiffness, *E*_*p*_, for Atg7^+/+^ SM22α-Cre^+^ (+/+ , open symbols) and Atg7^F/F^ SM22α-Cre^+^ (F/F, closed symbols) animals was determined at 80–120 (N) and 100–140 (H) mmHg in the presence of extracellular Ca^2+^ (**a**). Absolute change of *E*_*p*_ by removal of extracellular Ca^2+^ (**b**) at physiological (N) and high (H) pressures. Two-way ANOVA with pressure and genotype effect indicated and with Sidak’s multiple comparisons post-test. *p < 0.05 **p < 0.01 (*n* = 7)

Changes in vascular stiffness can be attributed to active, vascular tone-dependent factors and/or to passive, vessel wall intrinsic factors, which may occur independently of VSMC or EC reactivity [18]. Passive stiffness was determined by incubating the aortic segments in 0Ca^2+^ solution, reducing cytosolic Ca^2+^ and removal of basal active tone. To ensure that basal NO release did not interfere, 300 µM L-NAME was added to the organ baths. Two pressure ranges were investigated: 80–120 and 120–140 mmHg, named normal (N) and high (H) pressure. Whereas at physiological (N) pressures, diastolic diameter, compliance and *E*_*p*_ were not significantly different between Atg7^+/+^ SM22α-Cre^+^ or Atg7^F/F^ SM22α-Cre^+^ animals, aortic stiffness was significantly higher in Atg7^F/F^ SM22α-Cre^+^ animals at higher pressures (H) (Fig. 3a). Figure 3b shows the absolute change of stiffness by removal of external Ca^2+^ at normal and high pressures. Overall, there was a slight, but significant (*p* < 0.001) decrease in stiffness by removal of Ca^2+^, but the difference between both mouse strains at high pressure was not affected by Ca^2+^ removal. This finding indicates that passive aortic wall remodeling rather than differences in VSMC basal tone are responsible for the higher stiffness at elevated pressures.

### Defective autophagy in VSMCs enhances the thickness of the aortic vessel wall

**Fig. 4:**
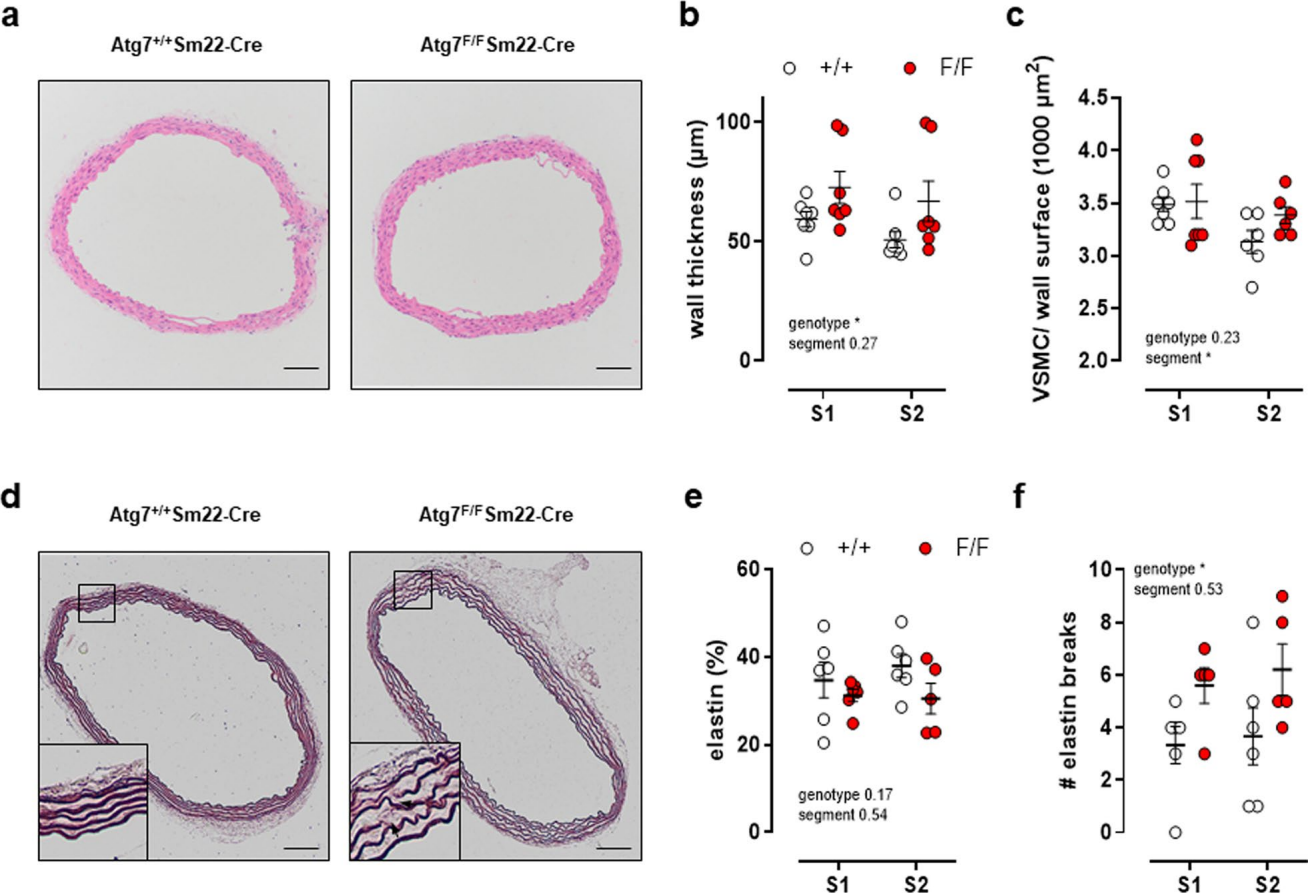
Defective autophagy in VSMCs enhances aortic vessel wall thickness. Hematoxylin/eosin staining (**a**) with quantification of vessel wall thickness (**b**) and number of VSMCs (**c**) as well as orcein staining (**d**) with quantification of the elastin amount (**e**) and number of elastin breaks (**f**) in aortic segments of Atg7^+/+^ SM22α-Cre^+^ (+/+) or Atg7^F/F^ SM22α-Cre^+^ (F/F) animals (*n* = 6–7). Measurements were performed in two segments (S1 and S2) of each aorta. Two-way ANOVA with segment and genotype effect indicated and with Sidak’s multiple comparisons post-test. *p < 0.05. Scale bar = 100 µm

The elevated passive stiffness of autophagy-deficient aortic segments can be caused by changes in aortic wall structure and the extracellular matrix. In particular, an increase in collagen content and/or a decrease in elastin content, possibly combined with elastin fragmentation, are associated with increased arterial stiffness [10, 19]. The medial wall thickness, as determined on H&E stained histological sections, indicated a significant increase in wall thickness by 25% in Atg7^F/F^ SM22α-Cre^+^ animals (Fig. 4a, b). The increase was not due to a raised number of VSMCs as there was no difference in the number of VSMCs per vessel wall surface (Fig. 4a, c). Histological analyses of the extracellular matrix components showed that the amount of collagen type I, III and IV and fibronectin was not significantly different between Atg7^F/F^ SM22α-Cre^+^ mice as compared to control mice (data not shown). However, in vessel segments of Atg7^F/F^ SM22α-Cre^+^ mice the amount of elastin fibers tended to decrease (Fig. 4d, e) and the number of elastin breaks tended to increase in aortic segments of Atg7^F/F^ SM22α-Cre^+^ mice (Fig. 4d, f).

### Defective autophagy in VSMC affects phenotype and focal adhesion

**Fig. 5:**
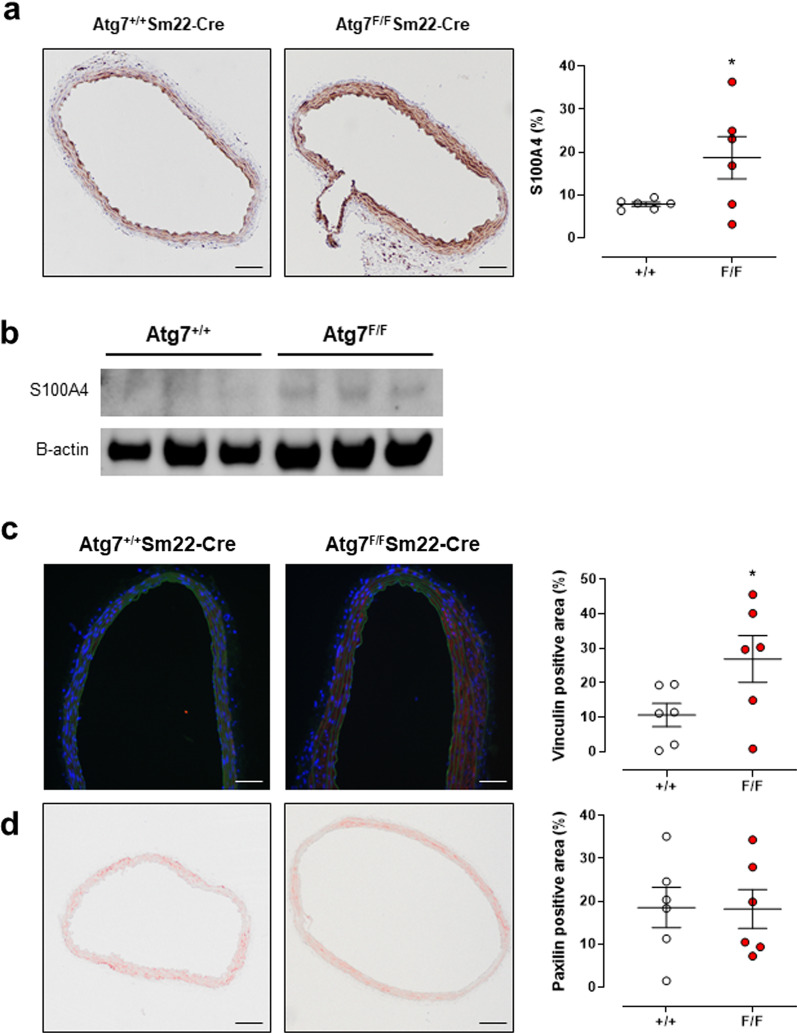
Defective autophagy in VSMCs increased S100A4, vinculin, but not paxillin. Immunohistochemical staining (**a**) and western blot analysis (**b**) of S100A4 in aortic segments of Atg7^+/+^ SM22α-Cre^+^ (+/+) or Atg7^F/F^ SM22α-Cre^+^ (F/F) animals (*n* = 6). Amount of vinculin (red, **c**) and paxillin (**d**) determined after immunohistochemical staining of aorta segments and Atg7^+/+^ SM22α-Cre^+^ (+/+) or Atg7^F/F^ SM22α-Cre^+^ (F/F) mice (*n* = 6). Two-way ANOVA on 2 segments per mice *p < 0.05 for genotype factor. Scale bar = 100 µm

VSMCs not only play a central role as active modulators of arterial stiffness, but are also crucial in the process of vascular remodeling. Indeed, it has been shown that in certain conditions VSMCs can undergo a phenotype switch from a quiescent contractile phenotype to a secretory phenotype. This dedifferentiation of VSMCs induces ECM secretion and hypertrophy, which can directly affect vascular stiffness [20]. The calcium binding protein S100A4, which was recently proposed as a marker of this phenotypic transition [21], was increased in the aorta of 2-month-old Atg7^F/F^ SM22α-Cre^+^ mice as compared to control mice, similarly as for 3.5-month-old Atg7^F/F^ SM22α-Cre^+^ mice [10] (Fig. 5a, b).

Elevated stiffness of the vascular wall is also modulated by the VSMC–extracellular matrix interaction. Indeed, focal adhesions, which form the connection between the VSMC cytoskeleton and the extracellular matrix, can be increased and may lead to higher arterial stiffness [22]. Therefore, we also measured the amount of vinculin, a focal adhesion molecule, in aortic segments of Atg7^+/+^ SM22α-Cre^+^ and Atg7^F/F^ SM22α-Cre^+^ mice (Fig. 5c). The amount of vinculin in the aorta of Atg7^F/F^ SM22α-Cre^+^ mice was significantly higher than in the aorta of control animals, in contrast to the focal adhesion protein paxillin, which was not different between the two groups (Fig. 5d).

Hitherto, most results obtained in 2-month-old Atg7^F/F^ SM22α-Cre^+^ mice were very similar to the results obtained in 3.5-month-old Atg7^F/F^ SM22α-Cre^+^ mice [9, 10], indicating that the effects of Atg7 knock-out on passive aortic biomechanics probably develop before the age of 2 months. The effects of intracellular Ca^2+^ decrease on basal tonus and the effects of autophagy deficiency on aortic diameter, were, however, age-dependent.

### Defective VSMC autophagy actively increases aortic stiffness

**Fig. 6:**
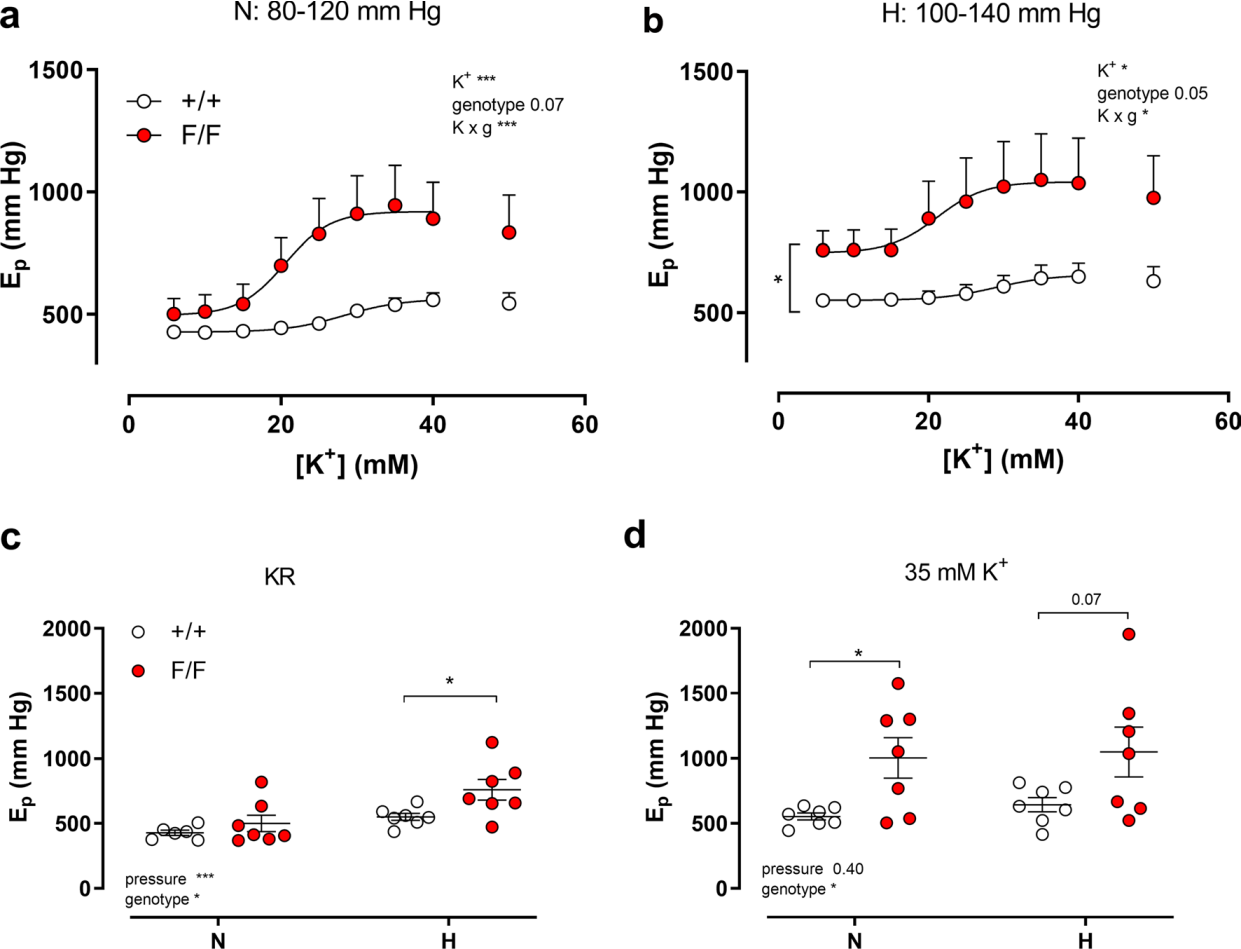
Depolarization-induced contractions increased arterial stiffness at low and high stretch pressure when autophagy was deficient in VSMCs. Concentration–response curves of stiffness increase elicited by K^+^ in aortic segments of Atg7^+/+^ SM22α-Cre^+^ (+/+) and Atg7^F/F^ SM22α-Cre^+^ (F/F) mice under physiological pressures (N, 80–120 mmHg, **a**) and elevated pressures (H, 100–140 mmHg; **b**) in the presence of L-NAME. *E*_*p*_ in unstimulated conditions (KR, **c**) and after depolarization with 35 mM K^+^ (**d**) at normal (N) and high (H) pressure. Two-way ANOVA with pressure and genotype effect indicated and with Sidak’s multiple comparisons test. *p < 0.05, **p < 0.01, ***p < 0.001 (*n* = 7)

Because the contractile status of VSMCs actively contributes to the isobaric biomechanical properties of the aorta [15, 18, 23], the effects of depolarization by extracellular K^+^ elevation on *E*_*p*_ were determined at physiological (N, 80–120 mmHg) and increased pressure (H, 100–140 mmHg) in the presence of 300 µM L-NAME. K^+^ concentration–stiffness curves at normal and higher distension pressures revealed higher stiffness of Atg7^F/F^ SM22α-Cre^+^ as compared to control aortic segments (Fig. 6a, b). Atg7^F/F^ SM22α-Cre^+^ segments were more sensitive to depolarization and EC_50_ values for K^+^ decreased from 27.4 ± 0.2 to 21.7 ± 1.7 mM (*p* < 0.01) at 80–120 mmHg and 29.6 ± 4.1 to 23.2 ± 4 mM (*p* < 0.01) at 100–140 mmHg. In Fig. 5c, d, the effects of autophagy deficiency on aortic biomechanical parameters are compared at normal pressure in KR (5.9 mM K^+^) and in depolarized (35 mM K^+^) conditions. Similarly to the previous experiments (Figs. 1c, 3a), *E*_*p*_ was not significantly different as compared to control mice in basal unstimulated conditions (5.9 mM K^+^) and at physiological pressures. However, when the distension pressure was increased, *E*_*p*_ values were significantly higher in Atg7^F/F^ SM22α-Cre^+^ mice (Fig. 6c). Contractions induced by depolarization with 35 mM K^+^ caused enhanced stiffening of the aortic segments of both mouse strains. The differences under stimulated conditions between Atg7^+/+^ SM22α-Cre^+^ and Atg7^F/F^ SM22α-Cre^+^ aortas were more pronounced at normal pressures (Fig. 6d).

**Fig. 7:**
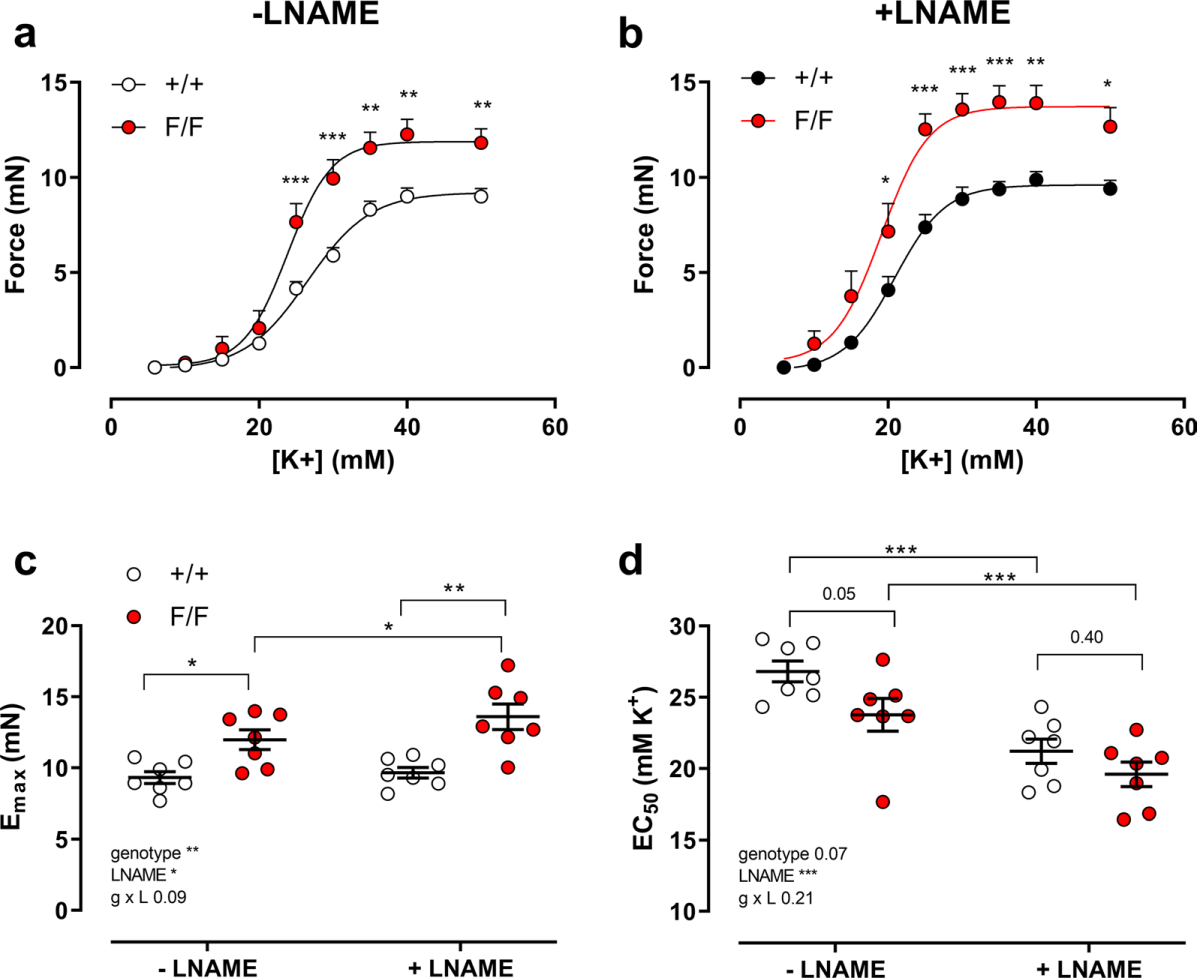
Defective autophagy in VSMCs increases maximum contraction and sensitivity to depolarization induced by increasing extracellular K^+^. Concentration–response curves of contractions elicited by K^+^ in aortic segments of Atg7^+/+^ SM22α-Cre^+^ (+/+) and Atg7^F/F^ SM22α-Cre^+^ (F/F) mice under control conditions (**a**) and in the presence of 300 µM L-NAME (**b**). Maximal force (**c**) and EC_50_ values (**d**) were determined in the absence and presence of 300 µM L-NAME from non-linear log(agonist)-response fits with variable slope (four parameter fits). Two-way ANOVA with genotype and L-NAME effect indicated and with Sidak’s multiple comparisons test. *p < 0.05, **p < 0.01, ***p < 0.001 (*n* = 7)

Our previous study with aortic segments indicated that in isometric conditions autophagy deficiency in mice of 3.5 months of age caused significant effects on vascular reactivity and calcium mobilization [9]. As expected from the results in Fig. 6, shorter duration of autophagy deficiency (6 week younger mice than in the previous study) also caused increased sensitivity of aortic segments to K^+^-induced depolarization in isometric conditions (Fig. 7). Maximal contractions elicited by 50 mM extracellular K^+^ concentration were significantly higher in aortic segments of Atg7^F/F^ SM22α-Cre^+^ as compared to Atg7^+/+^ SM22α-Cre^+^ mice, both in the absence and presence of 300 µM L-NAME to inhibit basal NO release (Fig. 7a–c). Furthermore, K^+^ concentration–response curves for Atg7^F/F^ SM22α-Cre^+^ mice displayed a leftward shift in the absence of L-NAME from 26.8 ± 0.7 to 23.1 ± 1.1 mM (*p* = 0.05) and a non-significant shift in the presence of L-NAME from 21.2 ± 0.9 to 20.1 ± 0.9 mM, indicating that the increased sensitivity of the Atg7^F/F^ SM22α-Cre^+^ aortic segments to depolarization is slightly NO-dependent (Fig. 7d).

It is remarkable that the leftward shift in the absence of L-NAME (± 3.7 mM) matched with the leftward shift at normal (± 5.7 mM) and higher (± 5.6 mM) pressure in the stiffness measurements of Fig. 6, although these were performed in the presence of L-NAME.

### Defective VSMC autophagy increases aortic stiffness depending on the mechanism of contraction

**Fig. 8:**
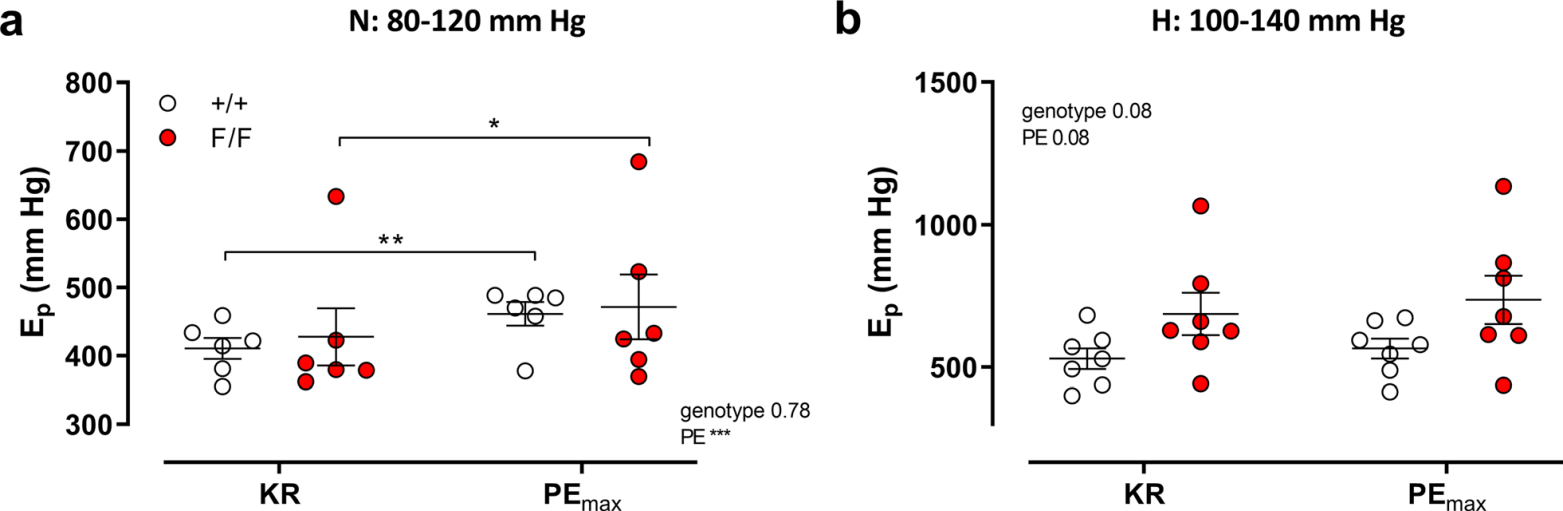
PE-induced contractions increased aortic stiffness at low and high stretch pressure independently of autophagy deficiency in VSMCs. *E*_*p*_ was measured in aortas of Atg7^+/+^ SM22α-Cre^+^ (+/+) and Atg7^F/F^ SM22α-Cre^+^ (F/F) mice at 80–120 mmHg stretch (N, **a**) and 100–140 mmHg stretch (H, **b**) in the absence (Krebs–Ringer, KR) and presence of 2 µM PE (PE_max_). Two-way ANOVA with pressure and genotype effect indicated and with Sidak’s multiple comparisons test. *p < 0.05, **p < 0.01, ***p < 0.001

To verify whether increased aortic stiffening at depolarized membrane potentials of the autophagy-deficient VSMCs is dependent upon the mechanism of contraction, aortic segments were maximally contracted with the *α*_1_-adrenoceptor agonist PE (2 µM) and aortic stiffness was measured at 80–120 and 100–140 mmHg. In contrast to depolarization with 35 mM K^+^, the decrease in diastolic diameter and compliance (data not shown) or the increase in aortic stiffness by 2 µM PE (Fig. 8a, b) was smaller than for depolarization with 35 mM K^+^ and the parameters were not significantly different between the two mouse strains at any pressure. Under PE-stimulated conditions, aortic segments of Atg7^F/F^ SM22α-Cre^+^ mice tended to be stiffer (but not statistically significant) than those of Atg7^+/+^ SM22α-Cre^+^ animals at high pressure (Fig. 8b).

**Fig. 9:**
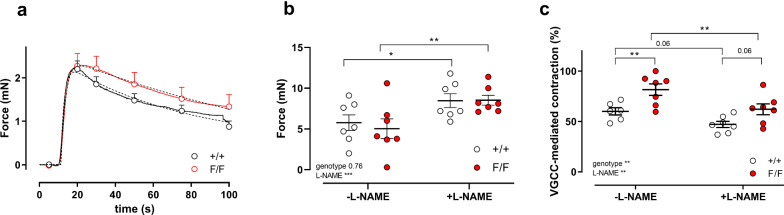
Defective autophagy in VSMCs affects IP3-mediated contractions and VGCC mediated tonic contractions by PE. IP3-mediated transient contractions of Atg^+/+^ SM22α-Cre^+^ (+/+) and Atg^F/F^ SM22α-Cre^+^ (F/F) aortic segments were elicited by 2 µM PE in 0Ca^2+^ conditions (**a**). Some data points of the contraction (shown as full line) are plotted ± SEM, whereas double exponential fit curves are shown as broken lines (parameters of curve fit in Table 1) (*n* = 5–7). Tonic contraction by 2 µM PE after addition of 3.5 mM Ca^2+^ to the 0Ca^2+^ solution (**b**) revealed no differences between +/+ and F/F aortic segments. Two-way ANOVA with L-NAME and genotype effect indicated (***p < 0.001) and with Sidak’s post hoc test. *p < 0.05, **p < 0.01 (*n* = 7). Relative amount of VGCC-mediated contraction to the total contraction by PE (**c**). Two-way ANOVA with L-NAME and genotype effect indicated (***p < 0.001) and with Sidak’s post hoc test; *p < 0.05, ***p < 0.001 (*n* = 7)

In isometric conditions, we determined phasic and tonic contractions of autophagy-deficient and competent aortic segments by addition of 2 µM PE. In the absence of extracellular Ca^2+^, PE elicited phasic contractions as shown in Fig. 9a. The area under the curve (Table 1) of inositol 1,4,5-triphosphate (IP_3_)-mediated contractions did not significantly differ between Atg7^F/F^ SM22α-Cre^+^ and Atg7^+/+^ SM22α-Cre^+^ mice. However, the time constant of relaxation (*τ*_relaxation_) was larger in Atg7^F/F^ SM22α-Cre^+^ as compared to Atg7^+/+^ SM22α-Cre^+^ mice, especially in the absence of L-NAME. The time constants of contraction (*τ*_contraction_) and the contraction/relaxation amplitudes were not significantly different between the two groups (Table 1).

**Table 1:** Parameters of phasic PE-induced contractions of Atg7^+/+^ SM22α-Cre^+^ (Atg7^+/+^) and Atg^F/F^ SM22α-Cre^+^ (Atg7^F/F^) aortic segments

	Atg7^+/+^	Atg7^F/F^	Atg7^+/+^ + L-NAME	Atg7^F/F^ + L-NAME
AUC (mN*s)	131 ± 19	121 ± 11	123 ± 15	139 ± 10
*τ*_contraction_ (s)	2.5 ± 0.7	3.2 ± 0.6	2.1 ± 0.2	3.2 ± 0.6
*τ*_relaxation_ (s)	26.8 ± 2.9	67.2 ± 17.2*	39.1 ± 12.9	41.9 ± 1.6
A_contraction_ (mN)	2.8 ± 0.2	2.7 ± 0.3	2.5 ± 0.2	2.5 ± 0.2
A_relaxation_ (mN)	− 1.7 ± 0.2	− 2.1 ± 0.3	− 1.8 ± 0.2	− 1.9 ± 0.4

Mean ± SEM (*n* = 5–7). Experiments were performed in the absence and presence of 300 µM L-NAME. Two-way ANOVA with Sidak post hoc test *p < 0.05 for genotype factor with *τ*_relaxation_, *p* = 0.054 for genotype factor with *τ*_contraction_

Tonic contractions by PE were evaluated after the re-addition of Ca^2+^ and were not significantly different between the two groups in the absence or presence of L-NAME (Fig. 9b). To determine the contribution of Ca^2+^ influx via voltage-gated Ca^2+^ channels (VGCC) and non-selective cation channels (NSCC) to the PE-induced tonic contractions, VGCC Ca^2+^ influx was completely blocked by addition of 35 µM diltiazem. Compatible with our previous observations at the age of 3.5 months, we found a larger contribution of VGCC to the contraction by PE as well in the absence as presence of L-NAME (Fig. 9c) in aortic segments of Atg7^F/F^ SM22α-Cre^+^ when compared with Atg7^+/+^ SM22α-Cre^+^ mice.

### Defective autophagy in VSMC increases NO in the aorta

**Fig. 10:**
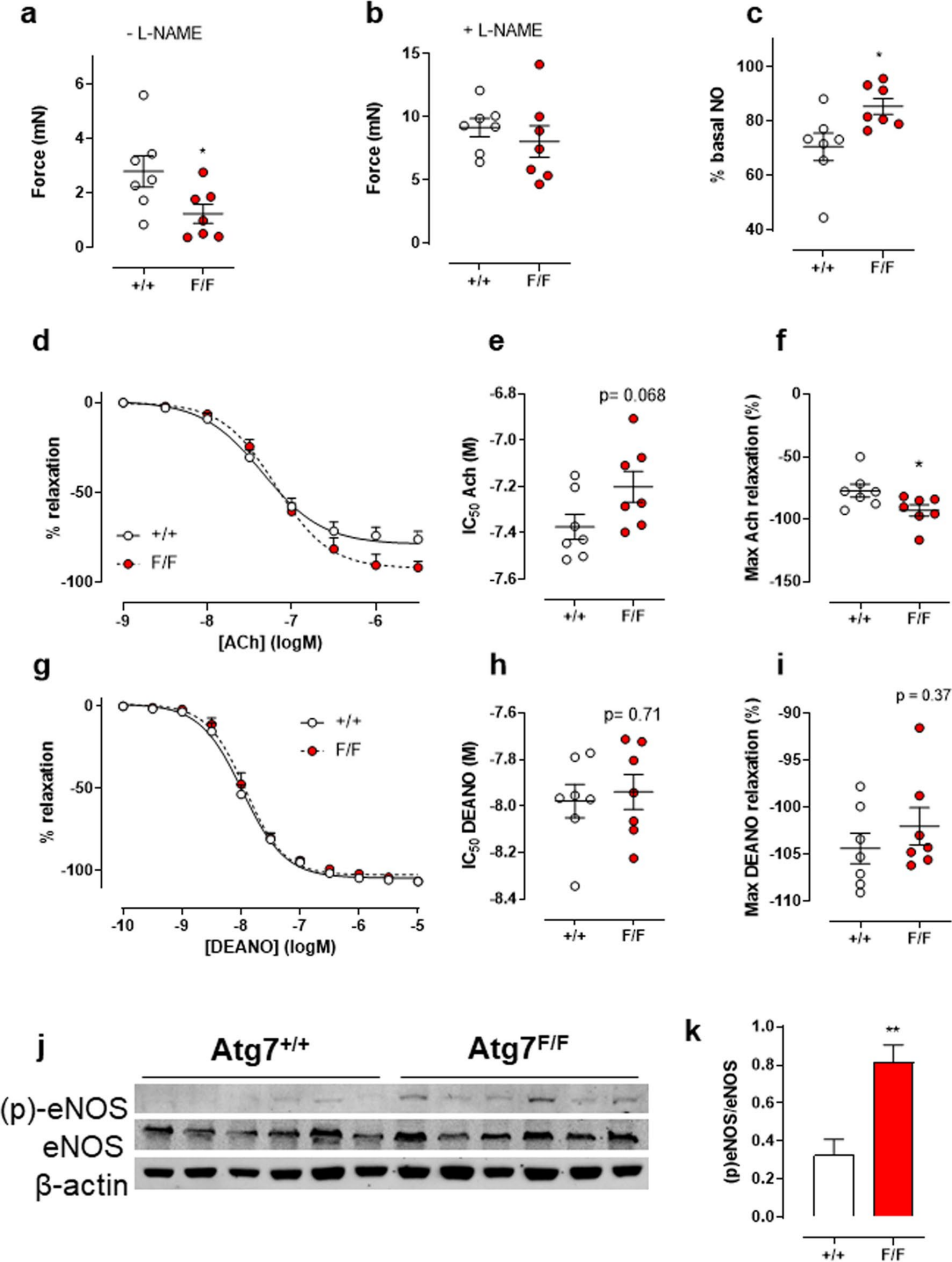
Defective autophagy in VSMCs increases the level of basal NO, stimulated NO release, and phosphorylated eNOS in the aorta without affecting exogenous NO-mediated relaxation. Force development after addition of 2 µM PE in aortic segments of Atg7^F/F^ SM22α-Cre^+^ (F/F) and Atg7^+/+^ SM22α-Cre^+^ (+/+) mice in the absence (**a**) or presence (**b**) of 300 µM L-NAME to calculate amounts of basal NO release (**c**). Relative concentration–response curves of relaxations elicited by acetylcholine (ACh) of Atg7^F/F^ SM22α-Cre^+^ (F/F) and Atg7^+/+^ SM22α-Cre^+^ (+/+) aortic segments (**d**), fitted IC_50_ (**e**) and maximal relaxation values (**f**). Relative concentration–response curves of relaxations elicited by DEANO of Atg7^F/F^ SM22α-Cre^+^ (F/F) and Atg7^+/+^ SM22α-Cre^+^ (+/+) aortic segments (**g**), fitted IC_50_ (**h**) and maximal relaxation values (**i**) (n = 7). Western blot (**j**) analysis of eNOS and (p)-eNOS with quantification relatively expressed to β-actin (**k**, **i**) (*n* = 6). Independent students *t* test. *p < 0. 05, **p < 0.01

Some experiments suggested that differences between the mouse strains were dependent on the presence or absence of L-NAME and, hence, of NO release. To verify whether basal NO release was affected by autophagy deficiency, isometric force development by 2 µM PE was measured before and after the addition of L-NAME (Fig. 10). In the absence of L-NAME, PE-induced contractions measured 1 h after mounting, were significantly lower in Atg7^F/F^ SM22α-Cre^+^ mice as compared to control mice (Fig. 10a). This difference, however, disappeared with the addition of L-NAME (Fig. 10b), indicating that aortic segments of Atg7^F/F^ SM22α-Cre^+^ mice have significantly higher levels of basal NO release (Fig. 10c).

NO-mediated relaxation of PE-pre-contracted aortic segments was determined in isometric conditions by inducing endothelium-dependent relaxation with acetylcholine (ACh) (Fig. 10c–e). Although a small non-significant decrease in ACh sensitivity was present in aortic segments of Atg7^F/F^ SM22α-Cre^+^ mice, a significant increase in the maximum relaxation occurred in mice with the VSMC autophagy defect. This effect is dependent on EC function since sensitivity and the maximal relaxation of the VSMCs to exogenous NO (DEANO) were not different between the two groups of mice (Fig. 10f–i). Finally, Western blot analysis confirmed that levels of eNOS phosphorylation in Atg7^F/F^ SM22α-Cre^+^ aortic segments were increased (Fig. 10j, k).

### In vivo vascular and cardiac parameters are not affected by autophagy deficiency in 2-month-old mice

Blood pressure and heart parameters were unaffected by autophagy deficiency in VSMCs at the age of 2 months (Table 2). There was no significant difference between Atg7^F/F^ SM22α-Cre^+^ mice and control mice in terms of aortic pulse wave velocity, the most important in vivo technique to measure arterial stiffness.

**Table 2:** Cardiac parameters, aPWV and blood pressure of Atg7^+/+^ SM22α-Cre^+^ (Atg7^+/+^) and Atg7^F/F^ SM22α-Cre^+^ (Atg7^F/F^) mice

	Atg7^+/+^	Atg7^F/F^
Ejection fraction (%)	76 ± 6 (5)	78 ± 3 (8)
Fractional shortening (%)	45 ± 6 (5)	46 ± 3 (8)
LV mass (mg)	78 ± 6 (5)	89 ± 13 (8)
Stroke volume (µL)	29 ± 3 (5)	29 ± 3 (8)
Systolic blood pressure (mmHg)	100 ± 4 (8)	99 ± 5 (10)
Diastolic blood pressure (mmHg)	68 ± 4 (8)	71 ± 5 (10)
Mean blood pressure (mmHg)	79 ± 4 (8)	80 ± 5 (10)
Pulse pressure (mmHg)	31 ± 2 (8)	29 ± 2 (10)
Pulse wave velocity (m/s)	2.0 ± 0.3 (6)	2.0 ± 0.3 (6)

Mean ± SEM (N) independent sample *t* test

## Discussion

Several lines of recent evidence indicate that autophagy stimulates VSMC survival, whereas reduced autophagy promotes age-related changes in the vasculature [2, 24]. Defective autophagy in VSMCs accelerates not only the development of stress-induced premature senescence, but also intimal thickening and atherosclerotic plaque formation [24]. The finding that VSMC senescence can promote atherosclerosis further illustrates that normal, adequate VSMC function is crucial in protecting the vessel wall against vascular disease.

### VSMC autophagy and arterial stiffening

**Fig. 11:**
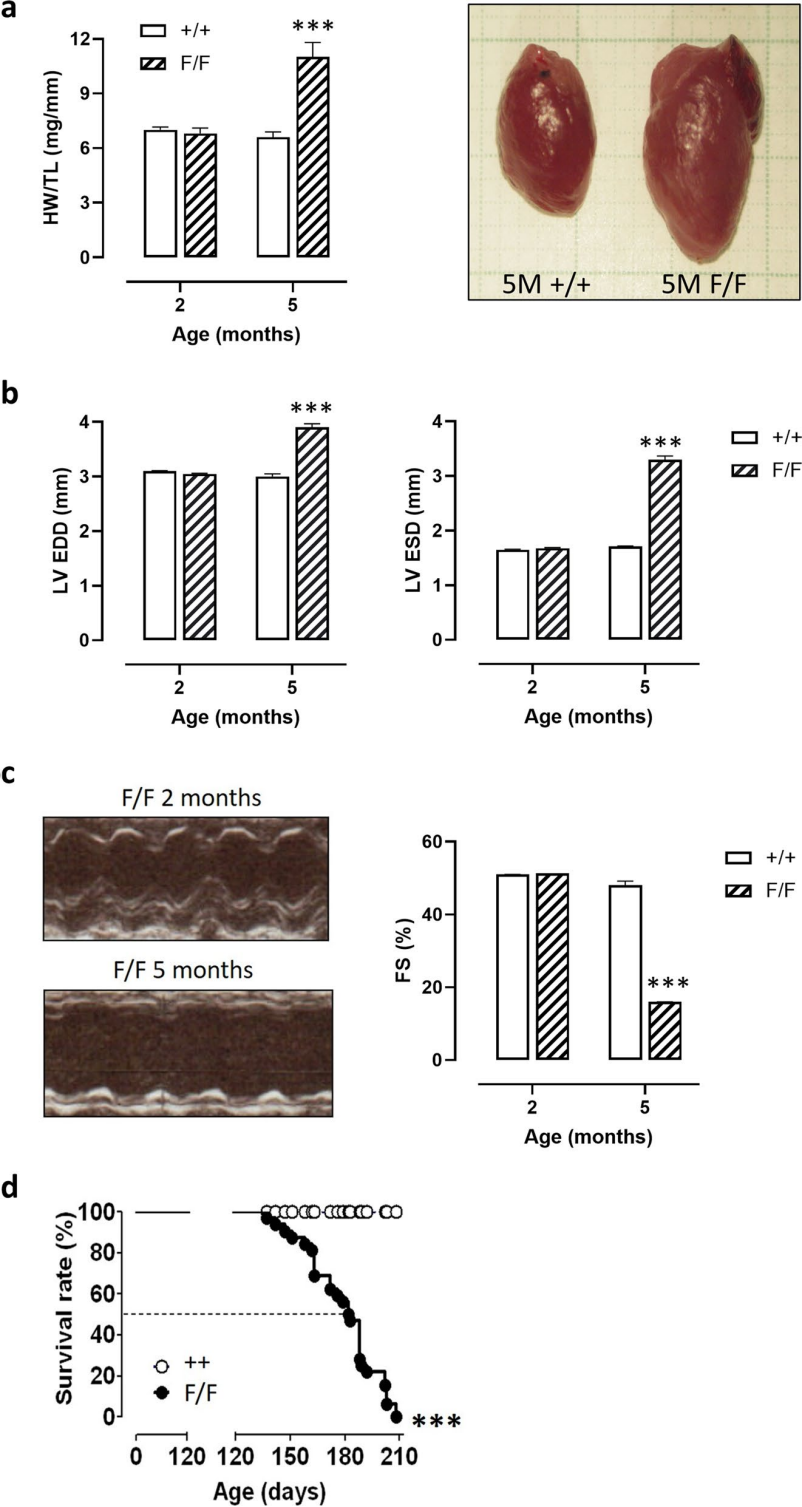
Atg7^F/F^ SM22α-Cre^+^ mice reveal heart failure and premature death starting at an age of 4.5 months. **a** Quantification of heart weight to tibia length ratio (HW/TL) in Atg7^+/+^ SM22α-Cre^+^ (+/+) mice and Atg7^F/F^ SM22α-Cre^+^ (F/F) at 2 and 5 months of age. Representative images of the heart from an Atg7^+/+^ SM22α-Cre^+^ and Atg7^F/F^ SM22α-Cre^+^ mouse at 5 months (5 M) of age are shown. **b** Quantification of left ventricular end-diastolic diameter (LV EDD) and left ventricular end-systolic diameter (LV ESD) in Atg7^+/+^ SM22α-Cre^+^ (+/+) and Atg7^F/F^ SM22α-Cre^+^ (F/F) mice at 2 and 5 months of age. **c** Fractional shortening (FS) in Atg7^+/+^ SM22α-Cre^+^ (+/+) and Atg7^F/F^ SM22α-Cre^+^ (F/F) mice at 2 and 5 months of age. Representative M-mode echocardiography images of Atg7^F/F^ SM22α-Cre^+^ (F/F) mice at 2 and 5 months of age are shown. **d** Kaplan–Meier survival curves of Atg7^+/+^ SM22α-Cre^+^ (+/+) and Atg7^F/F^ SM22α-Cre^+^ (F/F) mice. ***p < 0.001 versus +/+

**Fig. 12:**
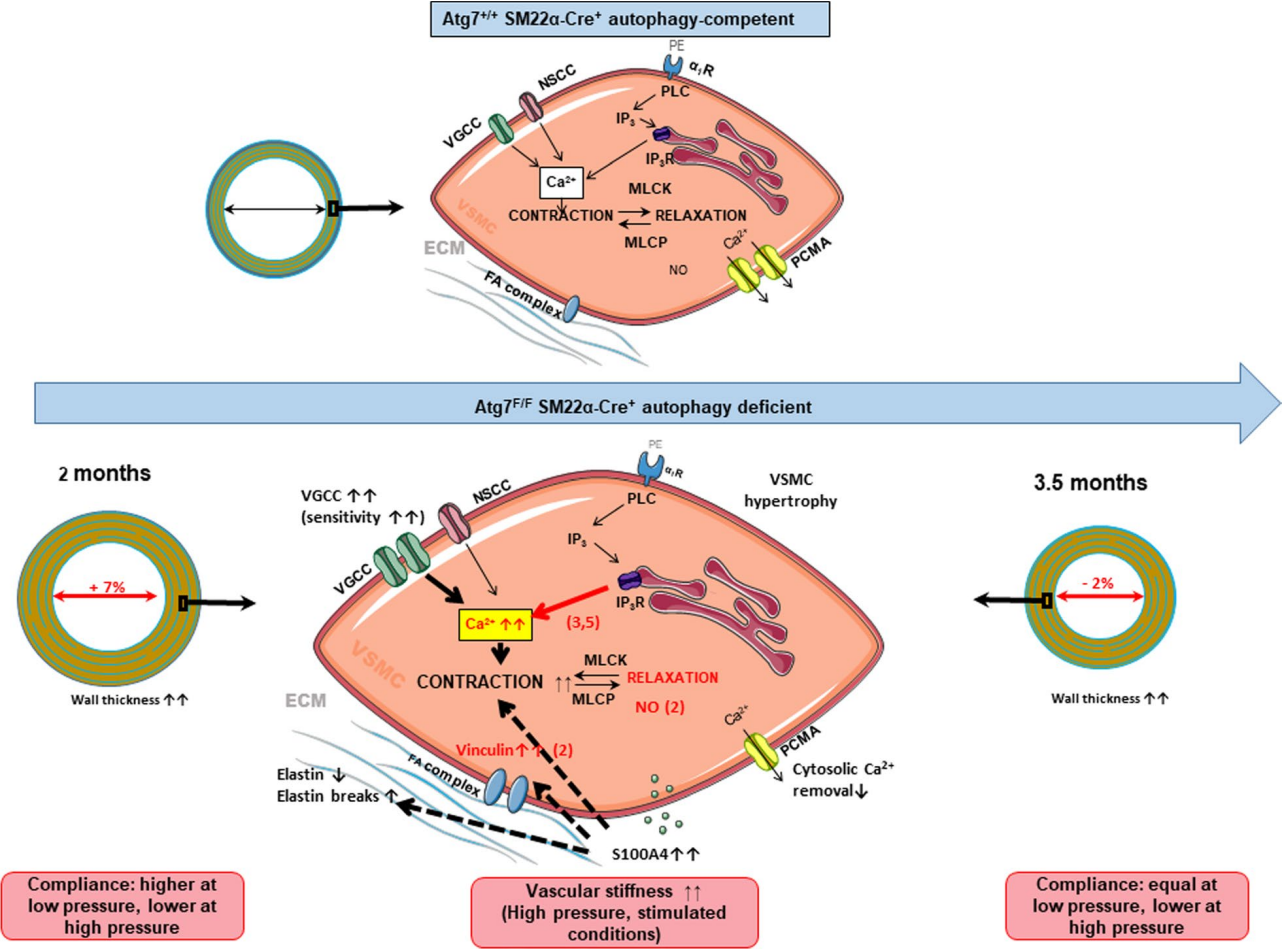
Schematic drawing of alterations in wall structure, biomechanics and smooth muscle cell function induced by autophagy deficiency in Atg7^F/F^ SM22α-Cre^+^ mice of 2 months (present study) and 3.5 months [9, 10] old as compared to their age-matched Atg7^+/+^ SM22α-Cre^+^ mice. Differences between 2 and 3.5 months are shown in red with between brackets the age of the mice. *VDCC* voltage-dependent Ca^2+^ channels; *NSCC* non-selective cation channels; *PE* phenylephrine; *α*_*1*_*R* *α*_1_-adrenoceptor; *PLC* phospholipase C; *IP*_*3*_*(R)* inositol trisphosphate receptor; *MLCK(P)* myosin light chain kinase (phosphatase); *PMCA* plasmalemmal Ca^2+^ pump; *SR* sarcoplasmic reticulum; *FA* focal adhesion; *ECM* extracellular matrix

Previous research revealed that knocking out the essential autophagy gene Atg7 in VSMCs by Cre-LoxP recombination (use of Atg7^F/F^ SM22α-Cre^+^ mice) has major effects on aortic VSMC function in 3.5-month-old mice [9, 10]. Indeed, autophagy deficiency in the VSMCs decreased aortic compliance, especially at higher than normal distension pressures, which was largely due to passive remodeling of the extracellular matrix. The question remains whether passive remodeling, which is often considered to be a longer-term adaptation of the vessel wall to acute changes in aortic performance, is time-dependently linked to the duration of autophagy knock-out. Therefore, the present study investigated biomechanical properties of the aorta of Atg7^F/F^ SM22α-Cre^+^ mice at the age of 2 months. Examining defective autophagy at 2 months has also an additional advantage. SM22α, used here to delete the essential autophagy gene Atg7 in VSMCs via Cre-LoxP technology, is transiently expressed in the heart during embryogenesis (between E8.0 and E12.5) [25] so that it is likely that cardiomyocytes in Atg7^F/F^ SM22α-Cre^+^ mice are partially autophagy defective. Because inhibition of autophagy in the heart induces age-related cardiomyopathy [26], Atg7^F/F^ SM22α-Cre^+^ mice develop severe heart failure (Fig. 11) associated with increased mortality starting at the age of 4.5 months. Accordingly, we cannot rule out the possibility that some changes in vascular function at 3.5 months of age are related to impaired cardiac function. We found that the altered isobaric aortic compliance and stiffness were already present for shorter term defective autophagy in aortic VSMCs, but we also observed autophagy deficiency duration-dependent parameters, which revealed a complex interplay between passive and active processes contributing to arterial compliance or stiffness (see also Fig. 12).

### Defective VSMC autophagy passively increases aortic stiffness

Similarly to the effects observed at 3.5 months [10], differences in aortic stiffness between 1.5 months younger Atg7^+/+^ SM22α-Cre^+^ and Atg7^F/F^ SM22α-Cre^+^ mice were highly pressure dependent, with the largest stiffening effect being present at higher distension pressures. The differences in basal, unstimulated conditions at high pressures are mainly driven by changes in the passive vascular stiffness since the incubation of the segment in 0Ca^2+^ to remove all VSMC tone did not affect the observed differences. In contrast to previous observations in autophagy-deficient aorta of older mice [9], but compatible with the lack of effect on aortic stiffness at 2 months at normal pressure, removal of external Ca^2+^ did not significantly change basal tone of aortic segments of 2-month-old Atg7^F/F^ SM22α-Cre^+^ animals mounted in isometric conditions (data not shown).

Similarly to the results at 3.5 months [10], an increase in elastin breaks and a slightly decreased elastin content (approximately − 5%) were observed, leading to differences in passive aortic stiffness between autophagy-competent and deficient aortas. This might explain the larger increase of stiffness in the aorta of mice with an autophagy deficiency in VSMCs at elevated distension pressures. By the loss of elasticity in the low pressure range (elastin reduction with elastin breaks) tensile force is redistributed to the stiffer collagen and, hence, stiffness increases. In this way, autophagy-deficient aorta resembles the aorta of elastin heterozygous mice (Eln^+/–^), in which about 60% of the normal elastin amount is present [27]. During normal vascular aging or in Eln^+/–^ mice, which are both characterized by reduced elastin content and/or increased elastin breaks, increased arterial stiffness was accompanied by mild cardiac hypertrophy, hypertension, narrowing (Eln^+/–^) or widening (ageing) of the large conductance vessels and an age-dependent attenuation of endothelial vasorelaxant function. Moreover, the Eln^+/–^ vessels were protected from an age-dependent alteration of *α*_1_ adrenoceptor-mediated vasoconstriction [27–29]. Hypertension of the Eln^+/–^ mice has further been attributed to permanent changes in vascular tone of resistance arteries: increased sensitivity to circulating AngII and attenuated endothelial function [30]. In Atg7^F/F^ SM22α-Cre^+^ mice, however, cardiac parameters and blood pressure were not affected by autophagy deficiency in VSMCs of mice at 2 or 3.5 months of age [10], which might be due to the less pronounced reduction in elastin content and an increase in elastin breaks and compensatory mechanisms in the circulation.

### Defective VSMC autophagy actively increases aortic stiffness

Although active and passive processes contribute to arterial stiffness and although VSMC contraction is an important active modulator of large artery compliance [18, 31], a clear distinction between these components is not always possible. The wall adaptations due to autophagy deficiency, mentioned before, cannot be ignored during contraction since the continuous connection of the elastin fibers to the contractile units in the VSMCs, which has been termed the “elastin-contractile unit” [32], is a prerequisite for performing contraction. The elastin content and function in the autophagy-deficient aorta is compromised, predicting a lower contractile function of the autophagy-deficient aortic VSMCs of Atg7^F/F^ SM22α-Cre^+^ mice. However, in isometric conditions, aortic segments of Atg7^F/F^ SM22α-Cre^+^ mice of 2 (this study) and 3.5 months [9] of age have increased sensitivity to depolarization-induced contraction, suggesting that this effect of autophagy deficiency on VGCC is age-independent between 2 and 3.5 months. The contractile Ca^2+^ content of the cytoplasm and the SR, on the other hand, seems to be age-dependent. Removal of extracellular Ca^2+^ reduced basal tonus significantly in aortic segments of 3.5-month-old, but not in segments of 2-month-old Atg7^F/F^ SM22α-Cre^+^ mice. In contrast to the significantly higher phasic contractions of 3.5-month-old Atg7^F/F^ SM22α-Cre^+^ mouse aorta [9], phasic contractions of aortic segments of 2-month-old mice were not significantly increased, although they did display slower relaxation. This finding further indicates that increased VGCC activity precedes the effect of autophagy on SR expansion and Ca^2+^ storage, which is consistent with previous findings that the contractile SR Ca^2+^ store refilling is dependent on the activity of VGCC [33]. Therefore, we propose that the differential *α*_1_ adrenoceptor-mediated effects on the VSMCs of autophagy-competent and deficient mice are age-dependent or, more specifically, dependent on the duration of autophagy knock-out. At least in the aorta, this age-dependency may involve autophagy-sensitive SR Ca^2+^ release and removal pathways, initially acting on plasmalemmal Ca^2+^ pumps [9].

The interaction between passive (ECM) and active (VSMCs) wall components in regulating aortic compliance is complex, because ECM–VSMC interactions are crucial elements in the determination of arterial wall stiffness [19, 22]. Besides the “elastin-contractile unit” [32], as mentioned before, this connection occurs at focal adhesion (FA) sites where the VSMC cytoskeleton is linked to ECM components through integrin-based interactions [19]. This linkage permits adequate force transmission from the contracted VSMC to the vascular wall via the extracellular matrix, thereby enabling stiffness development. Pharmacological inhibition of FA results in a more than 60% decrease in vascular stiffening, highlighting the importance of this mechanism as a regulator of arterial stiffness [22]. Interestingly, autophagy is also involved in cell adhesion. Inhibition of autophagy results in more and larger FA sites because autophagy is required for FA disassembly by degradation of paxillin [34, 35]. Surprisingly, in the present study, autophagy deficiency in VSMCs did not affect paxillin protein concentrations, but increased the focal adhesion protein vinculin. How autophagy deficiency affects the VSMC actin skeleton–focal adhesion–integrin–ECM axis, which definitely plays a role in active stiffening of arterial vessels [22, 31], is a topic for future research.

### Contractile Ca^2+^ mobilization and aortic stiffness

In control C57Bl/6J mice, VSMC stimulation by depolarization, in contrast to maximal stimulation with PE, has only limited effects on isobaric properties of the aorta [18]. In the present study, we found that, unlike a similar increase of isometric force (± 10 mN), maximal depolarization-induced stiffening of aortic segments with autophagy-deficient VSMCs was much more pronounced than maximal *α*_1_ adrenoceptor-mediated aortic stiffening. Stiffness increased by depolarization with 50 mM K^+^ (approximately 130 mmHg in Atg7^+/+^ SM22α-Cre^+^ aorta and approximately 450 mmHg in Atg7^F/F^ SM22α-Cre^+^ aorta) at normal distension pressure and was larger than by maximal PE concentrations (approximately 50 mmHg in the aortic segments of both strains). For Atg7^+/+^ SM22α-Cre^+^ aorta, this may be due to age-dependent effects of PE on aortic stiffness, because at 3.5 months, *E*_*p*_ increased by ± 450 mmHg by 2 µM PE at normal pressure [10], but for Atg7^F/F^ SM22α-Cre^+^ aortas, age-independent factors contribute. Among these factors different intracellular Ca^2+^ mobilization between autophagy-competent and deficient VSMCs may play a role.

### S100A4 and autophagy deficiency

We wondered how autophagy deficiency in the aortic VSMCs resulted in the reduction of the elastin content? Autophagy deficiency may induce a VSMC phenotype switch from the contractile to the synthetic phenotype and alter ECM synthesis. As a result, collagen synthesis and production of MMPs, which degrade elastin, may increase. In the present and former study [10], we did not observe any increased collagen synthesis, but we found that the Ca^2+^ binding protein S100A4 was increased, on average, in Atg7^F/F^ SM22α-Cre^+^ aortic segments. Although S100A4 is known to be upregulated during a VSMC phenotype transition, it is possible that a complete phenotype transition is not present because it has been described that autophagy is necessary for a phenotype modulation via the removal of contractile components [7, 13, 36]. However, there are conflicting data suggesting that autophagy inhibition induces phenotype modulation [8, 37, 38]. Contractile and synthetic VSMCs reflect two ends of an entire spectrum with intermediate phenotypes also present [39]. In addition, a clear distinction has to be made between extracellular and intracellular S100A4 [40]. While it is mainly the extracellular S100A4 that is related to VSMC phenotype transitions [21, 41], the intracellular domain is linked to cell adhesion and matrix remodeling [42, 43]. Increase of S100A4 in Atg7^F/F^ SM22α-Cre^+^ VSMCs, together with previous results by Grootaert et al. [8], which showed increased ligation-induced MMP9 activity in Atg7 defective VSMCs, indicate that matrix remodeling could be explained through S100A4 regulated MMP upregulation [9, 10, 14, 18, 23, 44]. All these parameters are affected by autophagy deficiency in VSMCs, albeit no distinction between extracellular and intracellular S100A4 was made.

Remarkably, in cancer cell lines S100A4 interacts with cytoskeletal proteins as well as with focal adhesion proteins such as Talin [43]. Therefore, it is conceivable that an increase in focal adhesion mediates the active component in the increased vascular stiffness at 2 months of age observed in the present study. S100 proteins may be involved as well. S100A4 expression is regulated by Ca^2+^ signaling pathways and in particular SR Ca^2+^ release since inhibition of SERCA with thapsigargin resulted in decreased S100A4 gene transcription [41].

### Defective VSMC autophagy affects endothelial function

Surprisingly, although only VSMC autophagy was defective in this animal model, isometric organ chamber experiments revealed that Atg7^F/F^ SM22α-Cre^+^ aortic segments demonstrated increased bioavailability of NO, whereas their sensitivity to exogenous NO was not affected. Besides basal unstimulated NO release, also receptor-mediated NO release was increased in aortic segments of Atg7^F/F^ SM22α-Cre^+^ mice, which was the result of enhanced EC function and increased phosphorylation of eNOS. Hence, increased production rather than decreased degradation must be responsible for this rise in basal and stimulated NO. The enhanced EC function was absent in Atg7^F/F^ SM22α-Cre^+^ aorta of 3.5-month-old mice and turned into endothelial dysfunction in 5-month-old mice (data not shown). Whether the EC function in 2-month-old Atg7^F/F^ SM22α-Cre^+^ mouse aorta was enhanced to compensate for altered VSMC function is still unclear. It has been described that S100 proteins are linked to NO release; addition of extracellular S100A4 to rat vascular endothelial cells partially restores decreased NO content after induction of oxidative stress by H_2_O_2_ [44]_._ Hence, increased synthesis and release of extracellular S100A4 from aortic VSMCs may contribute to the observed enhanced EC function in aorta of young Atg7^F/F^ SM22α-Cre^+^ mice.

### Autophagy and the vascular bed

Because vascular function differs between elastic and muscular arteries in health and disease [16, 23, 45], we expected that autophagy deficiency in VSMCs of elastic and muscular arteries causes vascular bed-specific functional changes. Comparison of aortic (this study) and femoral artery segments [11] of Atg7^+/+^ SM22α-Cre^+^ and Atg7^F/F^ SM22α-Cre^+^ mice of 2 months of age confirmed differential effects of autophagy deficiency in both blood vessels. In both vessels, autophagy deficiency in their VSMCs caused enhanced sensitivity to depolarization. Whereas aortic segments displayed enhanced EC function and equal sensitivity to exogenous NO, the femoral artery of Atg7^F/F^ SM22α-Cre^+^ mice was more sensitive to exogenous NO with normal ACh-induced relaxation, which could be interpreted as a compromised endothelial cell function. Phasic contractions induced by α_1_-adrenoceptor stimulation were significantly enhanced in the femoral artery, but not in the aortic segments of the Atg7^F/F^ SM22α-Cre^+^ mice of the same age, thereby resembling phasic contractions of 3.5-month-old Atg7^F/F^ SM22α-Cre^+^ aortic segments [9]. It is conceivable that autophagy deficiency in VSMCs of muscular arteries represents a shifted (older) time frame when compared to elastic arteries of mice of the same age.

### VSMC autophagy and in vivo cardiovascular parameters

Similarly to our observations at 3.5 months of age, in vivo measurements of arterial stiffness by abdominal aortic aPWV did not reveal any differences between Atg7^+/+^ SM22α-Cre^+^ and Atg7^F/F^ SM22α-Cre^+^ mice. Also the different cardiac parameters were not affected by autophagy deficiency in SMCs. The lack of effects on PWV was not surprising since most differences between Atg7^+/+^ SM22α-Cre^+^ and Atg7^F/F^ SM22α-Cre^+^ mice were observed at higher than normal pressures or under stimulated (depolarized) conditions, hence differentiating the ex vivo from the in vivo situation. Furthermore, in vivo measurements of aPWV are affected by numerous parameters (e.g., neuro-humoral factors and blood pressure). In addition, our measurements of aPWV were done under anesthesia, which by itself has an impact on vascular stiffness [46, 47].

### Conclusions

Using 3.5-month-old C57BL/6J mice, we previously demonstrated that autophagy deficiency in VSMCs affects VSMC contraction and cellular homeostasis with significant effects on vascular reactivity and calcium homeostasis [9, 10]. In the present study, we may conclude (based on a comparison between 3.5- and 2-month-old Atg7^F/F^ SM22α-Cre^+^ mice) that shorter term (2 months) autophagy deficiency leads to acute alterations in the vessel wall, including a higher segment diameter at 80 mmHg (+ 7% versus − 2% at 3.5 months), normal baseline tonus (versus increased), unchanged IP_3_-mediated phasic contractions (versus enhanced), and enhanced endothelial cell function (versus normal), indicating that autophagy deficiency in VSMCs at an early age initiates compensatory mechanisms to maintain circulatory homeostasis.

In Fig. 12, we compared different histological and physiological parameters between Atg7^F/F^ SM22α-Cre^+^ mice of 2 and 3.5 months old with respect to age-matched Atg7^+/+^ SM22α-Cre^+^ mice. Most of the histological parameters were similarly changed in Atg7^F/F^ SM22α-Cre^+^ mice of 2 and 3.5 months old. Autophagy deficiency in VSMCs of the aorta promoted aortic stiffening by elastin degradation and elastin breaks, especially at higher distension pressures. This occurred together with increased sensitivity of the VSMCs to depolarization and a larger contribution of VGCC mediated Ca^2+^ influx to *α*_1_ adrenergic contractions. Hence, all these phenomena occurred before the age of 2 months. Compared with autophagy-deficient aortic VSMCs of 3.5-month-old mice, autophagy deficiency of shorter duration (2 months) did not affect baseline tonus upon removal of extracellular Ca^2+^ or IP_3_-mediated phasic contractions, which displayed only slower relaxation, and EC function was enhanced. These latter processes are, therefore, dependent on the duration of autophagy deficiency in the VSMCs of Atg7^F/F^ SM22α-Cre^+^ aorta.

Unlike the age-dependent changes with autophagy deficiency in the VSMCs, the fact that in vivo parameters (cardiac function, blood pressure and aPWV) were not affected in Atg7^F/F^ SM22α-Cre^+^ mice of 2 and 3.5 months, further indicated that autophagy deficiency in the VSMCs of the aorta induces compensatory processes to maintain circulatory homeostasis. These processes may involve vessel wall remodeling, as well as adapted contractile behavior, and confirms the enormous capacity of the circulatory system for accommodating to changes induced by autophagy deficiency in VSMCs of the aorta [48].

## Data Availability

The datasets generated during and/or analyzed during the current study are available from the corresponding author on reasonable request. All data are available on demand.

## References

[1] De Meyer GRY, Grootaert MO, Michiels CF, Kurdi A, Schrijvers DM, Martinet W (2015). Autophagy in vascular disease. Circ Res.

[2] De Munck DG, De Meyer GRY, Martinet W (2020). Autophagy as an emerging therapeutic target for age-related vascular pathologies. Expert Opin Ther Targets.

[3] Nair S, Ren J (2012). Autophagy and cardiovascular aging: lesson learned from rapamycin. Cell Cycle.

[4] Nakamura S, Yoshimori T (2018). Autophagy and longevity. Mol Cells.

[5] Harvey A, Montezano AC, Touyz RM (2015). Vascular biology of ageing-Implications in hypertension. J Mol Cell Cardiol.

[6] Touyz RM, Alves-Lopes R, Rios FJ, Camargo LL, Anagnostopoulou A, Arner A, Montezano AC (2018). Vascular smooth muscle contraction in hypertension. Cardiovasc Res.

[7] Salabei JK, Cummins TD, Singh M, Jones SP, Bhatnagar A, Hill BG (2013). PDGF-mediated autophagy regulates vascular smooth muscle cell phenotype and resistance to oxidative stress. Biochem J.

[8] Grootaert MO, da Costa Martins PA, Bitsch N, Pintelon I, De Meyer GRY, Martinet W, Schrijvers DM (2015). Defective autophagy in vascular smooth muscle cells accelerates senescence and promotes neointima formation and atherogenesis. Autophagy.

[9] Michiels CF, Fransen P, De Munck DG, De Meyer GRY, Martinet W (2015). Defective autophagy in vascular smooth muscle cells alters contractility and Ca(2)(+) homeostasis in mice. Am J Physiol Heart Circ Physiol.

[10] De Munck DG, Leloup AJA, De Meyer GRY, Martinet W, Fransen P (2020). Defective autophagy in vascular smooth muscle cells increases passive stiffness of the mouse aortic vessel wall. Pflugers Arch.

[11] De Munck DG, De Moudt S, Roth L, De Meyer GRY, Martinet W, Fransen P (2020). Defective autophagy in vascular smooth muscle cells alters vascular reactivity of the mouse femoral artery. Front Physiol.

[12] De Moudt S, Leloup A, Van Hove C, De Meyer GRY, Fransen P (2017). Isometric stretch alters vascular reactivity of mouse aortic segments. Front Physiol.

[13] García-Miguel M, Riquelme JA, Norambuena-Soto I, Morales PE, Sanhueza-Olivares F, Nuñez-Soto C, Mondaca-Ruff D, Cancino-Arenas N, San Martín A, Chiong M (2018). Autophagy mediates tumor necrosis factor-α-induced phenotype switching in vascular smooth muscle A7r5 cell line. PLoS ONE.

[14] Michiels CF, Van Hove CE, Martinet W, De Meyer GRY, Fransen P (2014). L-type Ca^2+^ channel blockers inhibit the window contraction of mouse aorta segments with high affinity. Eur J Pharmacol.

[15] Leloup AJ, Van Hove CE, Kurdi A, De Moudt S, Martinet W, De Meyer GRY, Schrijvers DM, De Keulenaer GW, Fransen P (2016). A novel set-up for the ex vivo analysis of mechanical properties of mouse aortic segments stretched at physiological pressure and frequency. J Physiol.

[16] Fransen P, Van Hove CE, Leloup AJ, Schrijvers DM, De Meyer GRY, De Keulenaer GW (2016). Effect of angiotensin II-induced arterial hypertension on the voltage-dependent contractions of mouse arteries. Pflugers Arch.

[17] Di Lascio N, Stea F, Kusmic C, Sicari R, Faita F (2014). Non-invasive assessment of pulse wave velocity in mice by means of ultrasound images. Atherosclerosis.

[18] Leloup AJA, Van Hove CE, De Moudt S, De Meyer GRY, De Keulenaer GW, Fransen P (2019). Vascular smooth muscle cell contraction and relaxation in the isolated aorta: a critical regulator of large artery compliance. Physiol Rep.

[19] Lacolley P, Regnault V, Segers P, Laurent S (2017). Vascular smooth muscle cells and arterial stiffening: relevance in development, aging, and disease. Physiol Rev.

[20] Safar ME, Asmar R, Benetos A, Blacher J, Boutouyrie P, Lacolley P, Laurent S, London G, Pannier B, Protogerou A, Regnault V (2018). Interaction between hypertension and arterial stiffness. Hypertension.

[21] Chaabane C, Heizmann CW, Bochaton-Piallat ML (2015). Extracellular S100A4 induces smooth muscle cell phenotypic transition mediated by RAGE. Biochem Biophys Acta.

[22] Saphirstein RJ, Gao YZ, Jensen MH, Gallant CM, Vetterkind S, Moore JR, Morgan KG (2013). The focal adhesion: a regulated component of aortic stiffness. PLoS ONE.

[23] Leloup AJA, De Moudt S, Van Hove CE, Dugaucquier L, Vermeulen Z, Segers VFM, De Keulenaer GW, Fransen P (2018). Short-term angiotensin II treatment affects large artery biomechanics and function in the absence of small artery alterations in mice. Front Physiol.

[24] Grootaert MOJ, Moulis M, Roth L, Martinet W, Vindis C, Bennett MR, De Meyer GRY (2018). Vascular smooth muscle cell death, autophagy and senescence in atherosclerosis. Cardiovasc Res.

[25] Li L, Miano JM, Cserjesi P, Olson EN (1996). SM22 alpha, a marker of adult smooth muscle, is expressed in multiple myogenic lineages during embryogenesis. Circ Res.

[26] Taneike M, Yamaguchi O, Nakai A, Hikoso S, Takeda T, Mizote I, Oka T, Tamai T, Oyabu J, Murakawa T, Nishida K, Shimizu T, Hori M, Komuro I, Takuji Shirasawa TS, Mizushima N, Otsu K (2010). Inhibition of autophagy in the heart induces age-related cardiomyopathy. Autophagy.

[27] Wagenseil JE, Mecham RP (2012). Elastin in large artery stiffness and hypertension. J Cardiovasc Transl Res.

[28] Pezet M, Jacob MP, Escoubet B, Gheduzzi D, Tillet E, Perret P, Huber P, Quaglino D, Vranckx R, Li DY, Starcher B, Boyle WA, Mecham RP, Faury G (2008). Elastin haploinsufficiency induces alternative aging processes in the aorta. Rejuvenation Res.

[29] Thijssen DH, Carter SE, Green DJ (2016). Arterial structure and function in vascular ageing: are you as old as your arteries?. J Physiol.

[30] Osei-Owusu P, Knutsen RH, Kozel BA, Dietrich HH, Blumer KJ, Mecham RP (2014). Altered reactivity of resistance vasculature contributes to hypertension in elastin insufficiency. Am J Physiol Heart Circ Physiol.

[31] Gao YZ, Saphirstein RJ, Yamin R, Suki B, Morgan KG (2014). Aging impairs smooth muscle-mediated regulation of aortic stiffness: a defect in shock absorption function?. Am J Physiol Heart Circ Physiol.

[32] Milewicz DM, Trybus KM, Guo DC, Sweeney HL, Regalado E, Kamm K, Stull JT (2017). Altered smooth muscle cell force generation as a driver of thoracic aortic aneurysms and dissections. Arterioscler Thromb Vasc Biol.

[33] Leloup AJ, Van Hove CE, De Meyer GRY, Schrijvers DM, Fransen P (2015). Basal activity of voltage-gated Ca(2+) channels controls the IP3-mediated contraction by α(1)-adrenoceptor stimulation of mouse aorta segments. Eur J Pharmacol.

[34] Kawano S, Torisu T, Esaki M, Torisu K, Matsuno Y, Kitazono T (2017). Autophagy promotes degradation of internalized collagen and regulates distribution of focal adhesions to suppress cell adhesion. Biol Open.

[35] Sharifi MN, Mowers EE, Drake LE, Collier C, Chen H, Zamora M, Mui S, Macleod KF (2016). Autophagy promotes focal adhesion disassembly and cell motility of metastatic tumor cells through the direct interaction of paxillin with LC3. Cell Rep.

[36] Sun L, Zhao M, Liu A, Lv M, Zhang J, Li Y, Yang X, Wu Z (2018). Shear stress induces phenotypic modulation of vascular smooth muscle cells via AMPK/mTOR/ULK1-mediated autophagy. Cell Mol Neurobiol.

[37] An XR, Li X, Wei W, Li XX, Xu M (2018). Prostaglandin E1 inhibited diabetes-induced phenotypic switching of vascular smooth muscle cells through activating autophagy. Cell Physiol Biochem.

[38] Ni T, Gao F, Zhang J, Lin H, Luo H, Chi J, Guo H (2019). Impaired autophagy mediates hyperhomocysteinemia-induced HA-VSMC phenotypic switching. J Mol Histol.

[39] Rensen SS, Doevendans PA, van Eys GJ (2007). Regulation and characteristics of vascular smooth muscle cell phenotypic diversity. Neth Heart J.

[40] Brisset AC, Hao H, Camenzind E, Bacchetta M, Geinoz A, Sanchez JC, Chaponnier C, Gabbiani G, Bochaton-Piallat ML (2007). Intimal smooth muscle cells of porcine and human coronary artery express S100A4, a marker of the rhomboid phenotype in vitro. Circ Res.

[41] Kotnova AP, Lyanova BM, Dukhanina EA, Portseva TN, Ilyin YV, Georgieva SG, Stepchenko AG, Pankratova EV (2019). Thapsigargin, inhibitor of sarco-endoplasmic Ca(2+)-ATPase, effectively suppresses the expression of S100A4 protein in human breast cancer cell line. Dokl Biochem Biophys.

[42] Cao J, Geng L, Wu Q, Wang W, Chen Q, Lu L, Shen W, Chen Y (2013). Spatiotemporal expression of matrix metalloproteinases (MMPs) is regulated by the Ca^2+^-signal transducer S100A4 in the pathogenesis of thoracic aortic aneurysm. PLoS ONE.

[43] Indo HP, Matsui H, Chen J, Zhu H, Hawkins CL, Davies MJ, Yarana C, St Clair DK, Majima HJ (2015). Manganese superoxide dismutase promotes interaction of actin, S100A4 and Talin, and enhances rat gastric tumor cell invasion. J Clin Biochem Nutr.

[44] Meng X, Gao X, Zhang Z, Zhou X, Wu L, Yang M, Wang K, Ren H, Sun B, Wang T (2018). Protective effect and mechanism of rat recombinant S100 calcium-binding protein A4 on oxidative stress injury of rat vascular endothelial cells. Oncol Lett.

[45] Leloup AJ, Van Hove CE, Heykers A, Schrijvers DM, De Meyer GRY, Fransen P (2015). Elastic and muscular arteries differ in structure, basal NO production and voltage-gated Ca(2+)-channels. Front Physiol.

[46] Sehgel NL, Vatner SF, Meininger GA (2015). “Smooth muscle cell stiffness syndrome”-revisiting the structural basis of arterial stiffness. Front Physiol.

[47] Leloup AJ, Fransen P, Van Hove CE, Demolder M, De Keulenaer GW, Schrijvers DM (2014). Applanation tonometry in mice: a novel noninvasive technique to assess pulse wave velocity and arterial stiffness. Hypertension.

[48] Wagenseil JE, Mecham RP (2009). Vascular extracellular matrix and arterial mechanics. Physiol Rev.

